# Autopsy characteristics of deaths due to pulmonary thromboembolism in northern and western denmark: a 10-year retrospective study

**DOI:** 10.1007/s12024-024-00922-4

**Published:** 2024-12-02

**Authors:** Martin Roest Christensen, Annesofie Bjerrum Larsen, Lene Warner Thorup Boel

**Affiliations:** 1 Department of Forensic Medicine, Aarhus University, Palle Juul-Jensens Boulevard 99, Aarhus N, 8200 Denmark; 2 Aarhus Medical School, Faculty of Health, Aarhus University, Vennelyst Boulevard 4, Aarhus C, 8000 Denmark

**Keywords:** Pulmonary thromboembolism, Autopsy, Obesity, Body mass index, Cause of death

## Abstract

Because pulmonary thromboembolism (PTE) has an inherent high risk of sudden and unexpected death, this condition is a classic entity in forensic casework. The purpose of this study was to elucidate the characteristics surrounding deaths from PTE. We conducted a retrospective study from 2010 to 2019 at the Department of Forensic Medicine in Aarhus, Denmark. We recorded demographic characteristics, risk factors, comorbidities, and autopsy findings, including BMI. Furthermore, we contextualized the role of forensic autopsy in terms of determining the correct cause of death (COD).

Among the 3,572 autopsies, 58 had PTE as the main COD (1.6%), whereas only 0.3% of the deaths in the Danish COD registry were attributed to PTE in the same period. The decedents had a mean age of 52 years (range 19–87), and although the majority had preexisting comorbidities, approximately one-third died suddenly and unexpectedly. Additionally, more than half (35/58) of the decedents died in an out-of-hospital setting, and only a few of these (6/35) reported symptoms prior to death, underscoring the insidiousness of the condition. We identified a mean BMI of 32.7 among the decedents, with more than half of them (30/58) having a BMI ≥ 30.

In conclusion, obesity is a major risk factor for fatal PTE. The blurred clinical presentation of PTE underscores the importance of an autopsy to determine the correct COD, and with an increased autopsy rate, the true prevalence may well be higher.

## Introduction

Pulmonary thromboembolism (PTE) is a classic cause of death (COD) in forensic autopsy work, primarily because of the deceptive clinical presentation of a PTE, which results in sudden unexpected death. In Denmark, sudden unexpected death mandates a medicolegal inquest and a possible subsequent forensic autopsy. The insidious nature of PTE may be due to nonspecific clinical symptoms, such as dyspnea, coughing, and hemoptysis, which hinders diagnosis of the clinical condition [1, 2]. In addition, the diagnostic work-up for identifying a PTE [3, 4] may not be as straightforward as diagnosing a cerebrovascular catastrophe with a CT scan or a myocardial infarction with an EKG. Nevertheless, the incidence of PTE has increased markedly in recent decades (up to 83 per 100,000 adults per year), mainly because of the use of better and more precise diagnostic tools [5, 6]. However, PTE remains a critical, life-threatening condition, with a 30-day mortality rate of up to 11% at diagnosis [5, 7]. To further underscore the seriousness of the condition, current guidelines recommend the initiation of treatment within 24 h, even in hemodynamically stable patients with a low degree of clinical suspicion of PTE [8].

Although clinicians have become better at diagnosing PTE in living patients, a correct assessment of PTE as a COD without an autopsy still faces challenges. Several studies have repeatedly demonstrated the importance of an autopsy when determining a correct COD, even in deaths in the intensive care unit, where several clinical diagnostic tools and modalities (CT- and MRI-scans, echocardiography, etc.) are readily available [9, 10].

Major clinical and environmental risk factors for developing a PTE as a result of deep vein thrombosis (DVT) include advanced age, male sex, obesity, immobilization, major surgery, and cancer [5, 6, 11]. The multitude of different risk factors may contribute to the difficulty of a proper diagnosis of PTE ante mortem as well as the ability to state a correct COD in cases where an autopsy was not performed. Additionally, several genetic variations related to the coagulation cascade are associated with an increased risk of developing DVT and potentially a subsequent PTE. Mutations related to Factor V Leiden (FVL) are likely the best known, but genetic modulations related to Factor XIII, Beta fibrinogen (FGB), and others also have a thrombophilic predisposition [12].

Continuous elucidation of risk factors and clinical symptoms, as well as underscoring the insidious clinical presentation of PTE, is important for clinicians to correctly diagnose and treat PTEs. Furthermore, because of the indistinct clinical picture of PTEs, the role of a forensic autopsy is pivotal in determining a correct COD and provide the best possible COD statistics to aid prophylactic measures.

In this study, we not only highlight risk factors related to fatal PTE but also underscore the importance of a forensic autopsy in the correct determination of COD in cases of sudden death.

## Materials and methods

This work was a retrospective study based on autopsies performed at the Department of Forensic Medicine, Aarhus University, from 2010 to 2019. The department covers the majority of mainland Jutland, with approximately 2.2 million inhabitants. We searched the Department’s database and included cases in which PTE was determined to be the primary COD. We excluded cases in which the decedent was < 18 years of age. Each case file included police records with information on the circumstances leading up to the death, including testimony of the decedent’s general practitioner and next-of-kin as well as hospital records when applicable; an autopsy report; histological findings; and a postmortem toxicological analysis, with the latter two not being compulsory in the general case work-up.

Basic anthropometric data and variables regarding known comorbidities, antemortem symptoms, and postmortem findings were recorded for each case (Table 1). The study data were managed via REDCap electronic data capture tools hosted at Aarhus University [13, 14].

**Table 1:** Recorded variables from police records, hospital records, and autopsy reports

Basic characteristics and anthropometry	
Sex	
Age	
Height	
Weight	
BMI	
Comorbidities*	
Heart disease →	Hypertension
	Hypercholesterolemia
	Ischemic heart disease
Lung disease →	COPD
	Asthma
	Pneumonia
Cancer	
Major surgery	
Immobilization > 3 days	
Drug abuse	
Previous DVT	
Antemortem symptoms**	
Dyspnea	
Palpitations	
Fainting	
Cough and hemoptysis	
Dizziness	
Fever	
**Postmortem findings**	
Source of embolism	
Heart disease →	Atherosclerosis
	Myocardial fibrosis
Lung disease →	Emphysema
	Pneumonia

* In 50 out of 58 cases, the police records contained information from the deceased’s GP where the deceased had visited the GP within two years prior to death or from hospital records where the deceased had been admitted prior to death. ** In 47 out of 58 cases, the deceased was observed less than 24 h prior to death. Information on AM symptoms is based on eyewitness accounts. AM, antemortem; BMI, body mass index; COPD, chronic obstructive pulmonary disease; DVT, deep vein thrombosis; GP, general practitioner

The Shapiro‒Wilk test was used to assess the normality of the data. Parametric data were analyzed via Student’s *t* test. Logistic regression and one-way ANOVA were used to compare several means. Chi-square test and Fisher’s exact test were used for contingency tables, and the Bonferroni method was used for post hoc corrections for multiple comparisons. Statistical analyses were performed via R statistical software (v4.1.3; R Core Team 2022), and a *P* value < 0.05 prior to Bonferroni correction was considered significant.

## Results

During the study period, the department conducted 3,572 autopsies. Of these, 62 cases had PTE stated as the COD. However, two cases were excluded because the decedents were aged < 18 years, and two cases were excluded on further inspection of records because the COD was misclassified. Thus, the prevalence of PTE as a COD in our study period was 58/3,572 (1.6%).

Overall, we identified a slight male predominance (m: f ratio = 1.15:1), with an overall mean age of 52 years and a mean BMI of 32.7 kg/m^2^, with no significant sex differences (Table 2). With respect to comorbidities, 32 of the 58 decedents suffered from one or more types of disease (heart disease, lung disease and/or cancer). Only four of the deceased had previously had a DVT, and none had previously been diagnosed with a PTE.

**Table 2:** General characteristics of decedents with PTE as a COD. Neither variable significantly differed by sex (Student’s *t* test [age, BMI], chi-square test or Fisher’s exact test [comorbidities, postmortem findings], with Bonferroni correction *P* < 0.0045 was considered significant). BMI, body mass index; COD, cause of death; PTE, pulmonary thromboembolism

	Male	Female	Total
N	31	27	58
Mean age (range)	52.4 (28–84)	51.6 (19–87)	52 (19–87)
Mean BMI (95%CI)	32.4 (29.6–35.2)	33.1 (29.4–36.8)	32.7 (30.5–34.9)
*Comorbidities*			
Heart disease (yes/no)	11/18 (NA = 2)	8/18 (NA = 1)	19/36 (NA = 3)
Lung disease (yes/no)	9/20 (NA = 2)	8/18 (NA = 1)	17/38 (NA = 3)
Cancer (yes/no)	6/25	2/25	8/50
Major surgery	8/22 (NA = 1)	5/22	13/44 (NA = 1)
Immobilization	6/25	3/24	9/49
Drug abuse	12/12 (NA = 7)	4/18 (NA = 5)	16/30 (NA = 12)
*Postmortem findings*			
Source of embolism found (yes/no)	24/7	22/5	46/12
Heart disease (present/not present)	24/7	13/14	37/11
Lung disease (present/not present)	10/21	9/18	19/39

Almost one-third of our cohort had a history of illicit drug abuse. This finding underlines the influence of Danish law, which prompts the police to acquire a forensic autopsy in cases of deaths related to the use of illicit drugs. Concerning other risk factors, four of the 13 decedents who had undergone recent major surgery had also been immobilized. The records did not indicate whether these patients had received prophylactic antithrombotic treatment. Five decedents had been immobilized without prior surgery. However, almost half of the decedents in our study did not suffer from any of the medically related risk factors (heart disease, lung disease, and/or cancer). Six of these cases had undergone surgery, resulting in 20 decedents with no prior history of disease or surgery up to the time of death, underscoring the sudden onset of PTE.

Most of our decedents were defined as obese (BMI ≥ 30), with an equal number of normal weight and overweight people (BMI 18.5–25 and BMI 25–30, respectively). None of our cases were defined as underweight (BMI < 18.5) (Table 3). We found no significant difference in age among the three BMI categories. Similarly, the prevalence of cancer, heart disease, or lung disease did not significantly differ across BMI categories (data not shown).

**Table 3:** Sex and age distributions according to BMI categories. None of the variables significantly differed by BMI category (logistic regression [sex], one-way ANOVA [age], with Bonferroni correction *P* < 0.025 was considered significant). BMI, body mass index

		BMI	
	< 25	25–30	≥ 30
Sex (m: f)	5:6	6:5	16:14
Mean age (range)	52.6 (19–84)	55.5 (28–76)	49.0 (22–76)

**Fig. 1:**
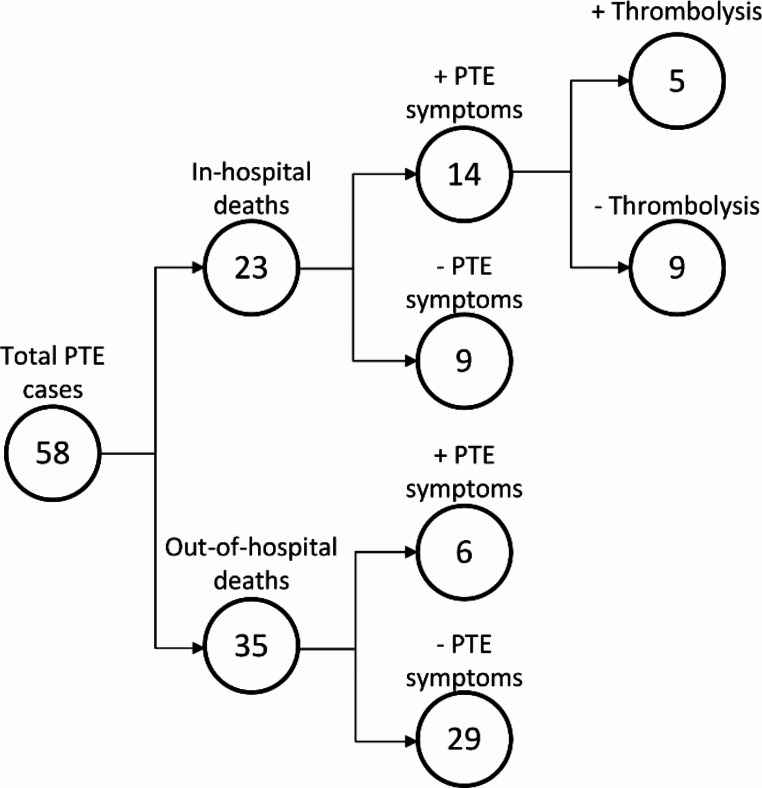
Distribution of death scene settings, symptoms of PTE, and whether antithrombotic treatment was administered

Most deaths occurred in an out-of-hospital setting (35/58), and only six of the decedents had reported symptoms of a PTE (dyspnea, fainting, palpitations, coughing and/or hemoptysis) prior to death (Fig. 1). Among the deaths that occurred in hospitals, clinicians suspected a PTE as the main cause of death in 14/23 cases. However, only in approximately one-third of these patients had received prophylactic antithrombotic treatment prior to death (Fig. 1), underscoring the high fatality of the condition.

In terms of postmortem findings, the origin of the PTE was determined at autopsy in most cases with 45 originating from a DVT in the leg and one originating from a thrombus in the venous plexus of the prostate. Regarding risk factors, almost two thirds of the cases had atherosclerosis and/or myocardial fibrosis and around one-third of the decedents had emphysema and/or pneumonia found at autopsy or at the subsequent microscopic examination (Table 2).

In 14 cases, the doctor performing the autopsy found it relevant to refer for an FVL mutation analysis. These cases were among the younger decedents our cohort (mean age 40.7 years), and only two were positive for an FVL mutation.

## Discussion

In this study, we investigated a decade of fatal PTEs at our institute with the advantages and limitations of forensic work.

Generally, forensic autopsy-based studies that have investigated fatal PTEs from the last decade have shown fairly heterogeneous cohorts with mean ages of decedents ranging from 40 to 67.8 years and a varying male: female ratio (0.5–1.5:1) [15–21]. Although the results of our study add to the heterogeneity of the overall forensic case work, our cohort constitutes the more centrist parts of the intervals. Further corroborating the heterogeneity, the prevalence of a fatal PTE ranges from 0.6 to 4.6% in autopsy cohorts [15–18, 20, 21]. This heterogeneity may reflect the basic limitations of forensic autopsy-based studies, with an inherent selection bias of cases determined by the police and/or authorities.

From a Danish perspective, annual reports from 2010 to 2019 on CODs from Statistics Denmark revealed that a total of 1,844 out of a total of 527,492 deaths (0.3%) were due to PTEs [22]. A recent Swedish study revealed that the decreasing prevalence of clinical autopsies overall reduced the value of the COD registry [23]. In Finland, where the forensic autopsy rate has remained stable at approximately 30% of all deaths for many years and has only recently experienced a decline, fatal PTEs constituted 2.3% of all deaths [24]. This finding may indicate that the true prevalence of fatal PTEs is underestimated in the Danish COD registry.

Based on several forensic autopsy-based studies that have examined fatal PTEs, the mean age of decedents appears to follow a bimodal distribution. For example, the present study, along with other studies, identified mean ages in the prime of the 50th decade [16, 19, 21], and others reported a mean age in the middle or ultimate part of the 60th decade [15, 18, 25]. Although general guidelines for referral for a forensic autopsy are similar across many countries, i.e., when death is sudden and/or unsuspected, certain details and national practices vary. Denmark has an inversely proportional relationship with advancing age and the probability of referral for a forensic autopsy [26], which may, to some extent, explain the relatively low mean age of our cohort. Although investigating national differences in national practices for referrals for a forensic autopsy is beyond the scope of the present study, we highlight the results from a study by Ylijoki-Sørensen et al., which revealed that broad referrals for a forensic autopsy in Finland had a marked impact on the low number of unknown CODs [27].

With respect to misclassifications of the COD in the clinical setting, an autopsy is valuable, especially with respect to PTEs. In the systematic review by Winters et al., PTE along with myocardial infarction, pneumonia, and Aspergillus infection constituted more than one-third of all Class I misdiagnoses according to the Goldman classification scheme, with PTE eclipsing the other three causes in the total number of studies identifying Class I and Class II misdiagnoses [9]. Winters et al. additionally proposed a mathematical model with a hypothetical value of 100% of decedents from a hospital intensive care unit undergoing an autopsy and reported an estimated incidence of 6.3% Goldman Class I misdiagnosis [9]. Furthermore, Ylijoki-Sørensen et al. reported that a systematic investigation with a medicolegal autopsy in all cases with an unclear COD only 2–3/1000 deaths would be coded as having an ill-defined or unknown COD compared with the present number of 41/1000 deaths [27].

Although a previous Danish study on acute PTE reported that cancer was the most prevalent risk factor [5], only approximately 1/6 of the deceased individuals in our cohort had a cancer diagnosis. This finding most likely reflects that deceased individuals with a known, potentially lethal disease—i.e., cancer—are less likely to be referred for medicolegal inquest and a potential subsequent forensic autopsy.

The impact of a patient’s BMI combined with the previously mentioned nonspecific clinical symptoms should raise the suspicion of a PTE. In accordance with other studies [15, 17, 19, 21, 25], the vast majority of our decedents were defined as overweight or obese. The prevalence of obesity in Denmark has increased during recent decades, and in 2021, almost one in every five adults (18.4%) could be classified as obese [28]. Although results from a forensic, retrospective study design rarely can be directly extrapolated to the general population, our rather consistent finding of fatal PTE in persons with a BMI > 25 and even more often with a BMI > 30 underscores the morbidity and mortality of obesity.

Many of the decedents in our study died in an out-of-hospital setting, which emphasizes the sudden and potentially fatal outcome of a PTE. Furthermore, only a few experienced symptoms prior to death, which is in line with previous studies [18, 21] and highlights the insidiousness of the condition.

Several genetic variations are associated with an increased risk of developing a DVT and, potentially, a subsequent PTE. A comprehensive autopsy-based study by Meißner et al. identified several genomic alterations related to an increased risk of DVT at different parts of the coagulation and fibrinolysis cascade. Specifically, genetic modulations related to FVL, Factor XIII, Beta fibrinogen, TFPI, and HIVEP1 were emphasized as points of interest in additional studies [12].

At our institute, we only referred cases for investigating FVL mutations in selected cases and only ¼ of our included cases were analyzed for this mutation. Fineschi et al. conducted a systematic, autopsy-based study investigating the prevalence of genetic mutations in cases of fatal PTE and reported that only 1/43 decedents had an FVL mutation [20]. We did not systematically investigate genetic mutations in our cohort, but the low prevalence of FVL mutation (2/58) may corroborate the conclusions of Fineschi et al. that only young decedents without major risk factors should be screened for genetic-based thrombophilia [20].

Although FVL mutation significantly increases the risk of a PTE [29], the mutation is associated with an even greater risk of developing a DVT. This discrepancy between the two entities in FVL mutations is dubbed the Factor V Leiden paradox. This paradox underscores the necessity of distinguishing the two conditions separately in thrombophilia studies and the importance of looking beyond FVL mutations in cases of fatal PTEs [12].

### Strengths and limitations

Because an autopsy is still regarded as the gold standard diagnostic tool for PTEs, an autopsy-based study into the characteristics of fatal PTEs constitutes a prime basis for the correct inclusion of cases. However, as with all studies based on forensic autopsies, the deceased individuals that are referred for a forensic autopsy are subject to inherent selection bias, which results in a small sample size. Because referral for a forensic autopsy and the age of the decedent are inversely related in Denmark [26], the current study focused on fatal PTEs, for which age is a known risk factor. Therefore, an unknown number of cases of fatal PTEs have not been referred for a forensic autopsy and ultimately were not included in our material. Finally, this study was retrospective; therefore, we relied on information extracted from police records, which precluded direct influence on the type of information that was available.

## Conclusion

In this study, we underscore how sudden and unexpected fatal PTEs can arise and how obesity is a major clinical risk factor associated with this condition. Furthermore, PTE as a COD in general is likely underestimated, and an increased focus on performing an autopsy in sudden and unexpected deaths, regardless of factors such as age, will elucidate the actual prevalence and circumstances surrounding the deaths, ultimately assisting the clinician to correctly diagnose PTEs in living patients.

## Key points

1. The majority of cases die in an out-of-hospital setting.
2. The nonspecific symptoms of PTEs highlight the insidiousness of the condition.
3. Obesity is a major risk factor for PTEs.
4. PTE as a cause of death is underdiagnosed, underscoring the importance of autopsy.
